# Spatiotemporal Signatures of Surprise Captured by Magnetoencephalography

**DOI:** 10.3389/fnsys.2022.865453

**Published:** 2022-06-13

**Authors:** Zahra Mousavi, Mohammad Mahdi Kiani, Hamid Aghajan

**Affiliations:** Department of Electrical Engineering, Sharif University of Technology, Tehran, Iran

**Keywords:** brain surprise, shift in belief, surprise decoder, oddball task, magnetoencephalography, decoding power, temporal/spatial MEG components

## Abstract

Surprise and social influence are linked through several neuropsychological mechanisms. By garnering attention, causing arousal, and motivating engagement, surprise provides a context for effective or durable social influence. Attention to a surprising event motivates the formation of an explanation or updating of models, while high arousal experiences due to surprise promote memory formation. They both encourage engagement with the surprising event through efforts aimed at understanding the situation. By affecting the behavior of the individual or a social group *via* setting an attractive engagement context, surprise plays an important role in shaping personal and social change. Surprise is an outcome of the brain’s function in constantly anticipating the future of sensory inputs based on past experiences. When new sensory data is different from the brain’s predictions shaped by recent trends, distinct neural signals are generated to report this surprise. As a quantitative approach to modeling the generation of brain surprise, input stimuli containing surprising elements are employed in experiments such as oddball tasks during which brain activity is recorded. Although surprise has been well characterized in many studies, an information-theoretical model to describe and predict the surprise level of an external stimulus in the recorded MEG data has not been reported to date, and setting forth such a model is the main objective of this paper. Through mining trial-by-trial MEG data in an oddball task according to theoretical definitions of surprise, the proposed surprise decoding model employs the entire epoch of the brain response to a stimulus to measure surprise and assesses which collection of temporal/spatial components in the recorded data can provide optimal power for describing the brain’s surprise. We considered three different theoretical formulations for surprise assuming the brain acts as an ideal observer that calculates transition probabilities to estimate the generative distribution of the input. We found that middle temporal components and the right and left fronto-central regions offer the strongest power for decoding surprise. Our findings provide a practical and rigorous method for measuring the brain’s surprise, which can be employed in conjunction with behavioral data to evaluate the interactive and social effects of surprising events.

## Introduction

The predictive coding framework (Rao and Ballard, 1999) postulates that the brain is constantly predicting its incoming sensory input. Past inputs are used by the brain to form prior knowledge while receiving the most recent input leads to updating of this belief in the Bayesian brain model (Friston, 2005; Doya et al., 2007; for a review see Kok and de Lange, 2015). An input different from what the brain has predicted will be surprising in that it generates a form of response measurable by brain imaging techniques. This surprise (or prediction error) has been quantified in the literature based on the expectation of a near-optimal observer who attempts to estimate the generative distribution of the input (Shannon, 1948; Baldi, 2002; Faraji et al., 2018). In addition, the quantified surprise has been widely shown to be reflected in the brain response, especially in the components of Event-Related Potentials (ERP) (Knill and Pouget, 2004; Strange et al., 2005; Mars et al., 2008; Friston, 2009; Itti and Baldi, 2009; Baldi and Itti, 2010; Meyniel et al., 2016; Seer et al., 2016; Modirshanechi et al., 2019; Musiolek et al., 2019). These studies underscore the importance and suitability of surprise to describe the neural activity in an uncertain environment.

A strong link exists between the concept of the brain’s surprise and social influence. Generation of a surprise signal by the brain instigates other functions which lead to eliciting the attention of the individual and influencing the course of cognitive processes involved in perception, memory formation, decision making, and engagement with the situation. Surprising events lead to engagement with the prevailing event through mechanisms such as attention and arousal (Russell and Barrett, 1999). When expectations about the sequence of events in a given context are violated, elevated attention levels are called for by the brain in order to find an explanation for the error. Surprising events hence attract attention and can lead to engagement with the source of surprise (Schützwohl, 1998; Horstmann, 2002; Itti and Baldi, 2009). The occurrence of surprise means that the brain’s model of the current event could not predict the particular instance recently observed and thus the model may need to be adjusted to make better predictions. Therefore, surprise changes what is believed and can hence influence its recipient by shaping both their perception and future behavior (Petty and Cacioppo, 1986; Loewenstein, 2019).

In addition, surprise is connected to high arousal experiences (Russell and Barrett, 1999). Efforts by the brain aimed at making sense of the situation promote memory for the event (Bradley et al., 1992). Another point is that people tend to share surprising contents with each other, rendering surprise to have the potential for large-scale social impact (Heath et al., 2001). Through setting an attractive engagement context, surprise influences the behavior of the individual or a social group and plays an important role in promoting personal and social change.

Studying the characteristics of surprise plays an important role in understanding how the mechanisms of attention and arousal, learning and memory formation, and decision to engage are formed in the brain. A remarkable observation is that the unpredictability of an instance in a sequence of stimuli which leads to a high value of surprise produces distinct brain signals in the process of eliciting the attention of the observer (Mars et al., 2008; Garrido et al., 2016; Rubin et al., 2016; Seer et al., 2016). In this context, surprise is often represented by a parameter that the brain attempts to minimize during the process of learning and perceptual inference (Schmidhuber, 2010; Roesch et al., 2012; Friston and Frith, 2015; Friston et al., 2017; Faraji et al., 2018).

In a recent study, it was discussed that surprise minimization not only plays a key role in the cognitive processes of a single agent, but also can be considered efficaciously in multi-agent frameworks to describe social phenomena like cooperation and social decision-making as well as explain the emergence of social rules for two agents (Hartwig and Peters, 2020). Importantly, Schwartenbeck et al. (2015) showed that in a simple binary choice setup, a surprise minimization paradigm could explain decision making better than utility maximization. In the context of predictive coding, the brain tries to avoid surprise to prevent stress, which can in long-term lead to heart disease, depression, and type 2 diabetes (Peters et al., 2017).

Shannon surprise (Shannon, 1948) has been widely used as a measure for quantifying surprise based on the likelihood of the data (Strange et al., 2005; Mars et al., 2008; Kolossa et al., 2015; Meyniel et al., 2016; Rubin et al., 2016; Seer et al., 2016; Modirshanechi et al., 2019). The more “unlikely” an input is, the more the value of its corresponding Shannon surprise will be. The Bayesian surprise differentiates the estimated generative distribution of the received stimuli before and after the arrival of each input. Therefore, it quantifies how the belief about the distribution of the input is “updated” or “shifted” after receiving each stimulus. This concept of surprise was introduced by Baldi (2002) and has been used thereafter by many researchers (Mars et al., 2008; Itti and Baldi, 2009; Baldi and Itti, 2010; Seer et al., 2016; Musiolek et al., 2019). Faraji et al. (2018) introduced an alternative quantification of surprise, named the confidence-corrected surprise, which reflects the “unexpectedness” (not unlikeliness) of the input by differentiating the estimated posterior distribution of the input with that of a naïve observer (who bases his model on the most recent input and a uniform prior) using the Kullback–Leibler (KL) divergence (Kullback, 1997; Cover, 1999).

Temporal components of MEG (Magnetoencephalography) records that represent surprise have not been as much investigated as EEG (Electroencephalography) data. Nevertheless, some studies have focused on how the violation of an expected event in a sequence of stimuli is reflected in the MEG response (Chait et al., 2007; Todorovic et al., 2011; Wacongne et al., 2011; Todorovic and de Lange, 2012; Strauss et al., 2015; Barascud et al., 2016; Heilbron and Chait, 2018). These studies include reports on the observation of mismatch components in the brain’s MEG response to unpredicted stimuli or novelty.

Previous surprise modeling studies mainly base their conclusion on a single component extracted from the EEG data, with the MMN (mismatch negativity) (Garrido et al., 2009; Lieder et al., 2013) or the P300 (Squires et al., 1976; Mars et al., 2008; Kolossa et al., 2013) or both (Ostwald et al., 2012) serving as the main components revealing the occurrence of surprise. Abnormal values in these components have also been proposed as biomarkers for cognitive disorders such as Schizophrenia and Alzheimer’s disease (Nieuwenhuis et al., 2005; Patel et al., 2005; Barcelo et al., 2006; Duncan et al., 2009), reflecting their importance not only in understanding the behavior of the normal brain in handling surprise, but also in the detection of a number of brain disorders. While such single component analysis simplifies the ensuing effort to develop an encoder or a decoder for the brain surprise, it ignores the possible contribution of other temporal components corresponding to different post-stimulus latencies.

Recent studies have proposed models using the entire temporal signals for decoding Shannon surprise (Maheu et al., 2019; Modirshanechi et al., 2019; Gijsen et al., 2021), assuming that the entire epoch of the response might be modulated by the statistical properties of the input sequence. We will take a similar approach in this paper and mine trial-by-trial MEG data to analyze how the entire epoch of the brain response reflects the prediction error and which collection of the temporal/spatial components provide optimal power for describing the brain’s surprise.

In a study by Modirshanechi et al. (2019), the density of significant temporal features for decoding Shannon surprise was compared in the middle and late segments of EEG data and no significant difference was observed between these two segments in terms of decoding surprise. Also, Maheu et al. (2019) conducted a study on MEG data with participants exposed to auditory sequences with different statistical regularities, and modeled the activity of the brain with Shannon surprise levels using several learning models. Gijsen et al. (2021) described the EEG dynamics of the somatosensory learning system in terms of its neural surprise signatures.

In the current study, aside from considering different concepts of surprise, the value of each of the temporal components is assessed and compared with others in MEG records of an auditory oddball task. Besides, analytical definitions are proposed for the early, middle, and late segments based on a method that partitions the response of each trial to three temporal segments based on the behavior of each segment in describing surprise. We compare the middle part of the recorded response and the late part in terms of reflecting the surprise of the brain. We aim to examine whether there is one temporal component or a subset of components that best describe each of the three mentioned surprise concepts. We also perform a sensor-level analysis to identify the best locations on the scalp to capture information about surprise from neural activities.

The repetition-break plot structure (Loewenstein and Heath, 2009) is one of the recipes proposed for eliciting surprise in studies on its social influence (Loewenstein, 2019). In computational frameworks for studying surprise based on measured brain signals, oddball experiments are employed in which repeated exposure to surprising instances of the stimuli allow for trial averaging and noise reduction. The current study focuses on binary oddball tasks and formulates its definition of surprise assuming a transition probability matrix that describes the generative distribution of the stimuli sequence (Meyniel et al., 2016). Considering the generative distribution as a Markov process, this transition probability matrix serves as sufficient statistics to describe the distribution. It was shown in Meyniel et al. (2016) that this assumption leads to a surprise value (prediction error) that is highly correlated with the P300 response. Also, Gijsen et al. (2021) showed that this first order transition probability is the best inference model in terms of goodness of fit to EEG data.

The paper sets forth comparative results for the mentioned surprise decoders, and statistically elaborates on the relative importance of the different channels/temporal components in decoding the three surprise concepts (Shannon, Bayesian, and confidence-corrected surprise which, respectively, represent the unlikeliness, updating, and the unexpectedness of the input) elicited by the stimuli. The results support the Bayesian learning assumption and provide evidence for predictive coding.

## Materials and Methods

**FIGURE 1:**
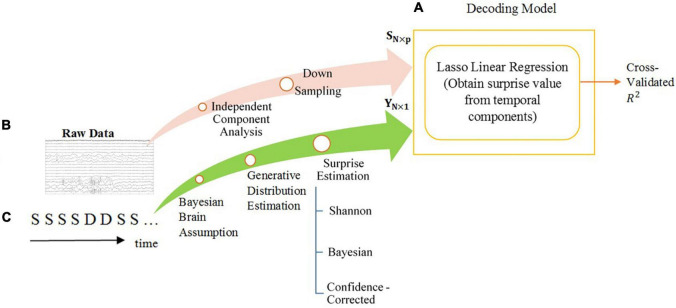
The overall diagram of the decoding model of temporal analysis. The steps are explained in the Section “Materials and Methods”. **(A)** The scheme of the decoding model and machine learning tools. The power of decoding is measured by the fraction of variance that is explained (*R*-squared). **(B)** The processes performed on the preprocessed MEG data to acquire features for regression. The feature matrix is shown by *S*_*N×p*_. The length of each feature is *p* and the number of features is *N*. **(C)** The surprise calculation module using the oddball sequence of stimuli, consisting of standards S and deviants D, as input and generating labels for training the regression model. The labels vector is shown as *Y*_*N×1*_.

Figure 1 provides an overview of the overall flow of data and the decoding approach used in our analysis.

### Dataset and Task

Our analysis is applied to a dataset consisting of MEG responses recorded in an auditory oddball task (Maheu et al., 2019). In this task, the standard and deviant stimuli were two different French syllables randomly drawn from a binomial distribution with the probability of the frequent syllable being 2/3 and that of the deviant syllable being 1/3. Each syllable lasted about 200 milliseconds and the interval between two successive stimuli was 1400 milliseconds. The data record consisted of one block of stimuli with around 405 trials.

Participants included 11 females and 9 males, aged between 18 and 25. The data of two subjects were removed because of their excessive head movements. To ensure that the participants paid attention to the task, they were asked every 12–18 trials to predict the next stimuli (being a standard or a deviant) using one of two buttons.

The brain activity was recorded by a 306 channels (102 magnetometers and 204 gradiometers) whole-head Elekta Neuromag MEG system using a sampling rate of 1000 Hz and a hardware-based band-pass filter of 0.1–330 Hz.

### Preprocessing of Data

The following preprocessing steps were performed on the raw data as reported by Maheu et al. (2019): Raw MEG data were corrected for between-session head movement and bad channels. Then, data were epoched between −250 ms to 1 s and were also cleaned from powerline and muscle and other movement artifacts. Trials containing muscle artifacts were detected using semi-automatic methods (based on the variance of signals across sensors and first order derivatives of signals over time) and removed. Then, a low-pass filter below 30 and a 250 Hz down-sampler was applied to the data. Eye blinks and cardiac artifacts were removed using ICA (Independent Component Analysis) (Bell and Sejnowski, 1995). Finally, the data was baseline corrected using a window of 250 ms before the stimulus onset. Similar to the earlier study (Maheu et al., 2019), the analysis was performed only on the data of the magnetometers using the EEGLAB toolbox (Delorme and Makeig, 2004).

For temporal analysis, in order to obtain independent sources of MEG record as features of the regression model, we performed ICA analysis (Bell and Sejnowski, 1995). We chose FastICA (Hyvärinen, 1999) for this data because of the high number of channels (102) which could render the InfoMax algorithm excessively slow. We ended up with an average (over subjects) of 69 independent components for the entire set of sensors using FastICA. We also considered the interval of [−200 ms, 600 ms] as the response period and reduced the number of samples by downsampling to 80 samples per epoch. We took each trial as a feature, so the number of features used for training was *N* ∈ [400, 409] (equal to the number of stimuli in the block which varied between the participants). We concatenated the vectors of independent components to make a longer vector which serves as the decoder input. Thus, the maximum dimension of each feature was around *p* = 80×69 = 5520 (equal to the number of time samples multiplied by the number of independent components). The superiority of using independent components instead of the data of the channels is that the resulting feature vectors contain lower dependencies between their elements.

For spatial analysis, for the recorded signal of each channel, we selected the interval of [−200 ms, 600 ms] as the response period and reduced the number of samples by downsampling to 80.

### Ideal Observer Model

A fundamental question in the Bayesian brain literature is how the brain learns the distribution of the sensory stimuli. The brain is assumed a near-optimal estimator of the probability of the input sequence based on a generative model with Bayesian inference (Mars et al., 2008; Daunizeau et al., 2010; Friston, 2012; Meyniel et al., 2016; Rubin et al., 2016; Modirshanechi et al., 2019). To be more precise, the brain uses a prior belief about the environment, and updates it after each stimulus arrives. In addition, in order to initialize the inference process, it is presumed that the brain begins with the assumption of equally probable input types despite exposure to any possible previous blocks of stimuli (Strange et al., 2005; Harrison et al., 2006; Bestmann et al., 2008; Meyniel et al., 2016).

Here, two crucial questions to ponder on are what exactly constitutes the statistics that the brain attempts to learn from the recent history of observations, and what mechanism it employs to arrive at an optimal estimate of this probability.

### Transition Probabilities

In an oddball experiment, each stimulus can be denoted by a binary random variable *x^i^* for *i* = 1,…,*T*, where *T* is the length of the stimuli sequence. We consider *x^i^* = 0 if the *ith* stimulus is a standard and *x^i^* = 1 otherwise. This variable follows a Binomial distribution with parameters *p*_*0*_ and *p*_1_ = 1−*p*_*o*_ as the probabilities of the standard and deviant stimuli, respectively. Based on the hypothesis that the sequence of items has been generated by a “Markovian” generative process, the sequence can be modeled by the probabilities of transition between the stimuli types. For a binary oddball sequence, the transition probabilities can be stated as a 2×2 matrix, which can be estimated by counting the number of successive transitions (Meyniel et al., 2016). It has been demonstrated that utilizing the transition probability matrix for describing the stimuli sequence statistically outperforms the single-parameter approach to describe the brain’s response (Meyniel et al., 2016).

For a binary oddball sequence, the definition of the model parameter θ can be stated in the form of a 2×2 matrix:


$$\theta ≜\left[\begin{array}{cc} p_{0|0} & p_{0|1}\\ p_{1|0} & p_{1|1} \end{array}\right],$$

where *p*_*a—b*_ is the probability of transition from stimulus type *b* to stimulus type *a*. Since the sum of each column of this matrix is equal to 1, we can reduce the model parameter’s definition to a vectorθ :


$$\theta =\left[\begin{array}{c} \theta_{0|1}\\ \theta_{1|0} \end{array}\right]=\left[\begin{array}{c} p_{0|1}\\ p_{1|0} \end{array}\right].$$

Based on this definition, the likelihood of a sequence of observations **X***^j^*with a length *j* will be:


(1) p(Xj|θj)=0.5(θ0|1j(1-θ0|1j)n1|1jn0|1j)(θ1|0j(1-θ1|0j)n0|0jn1|0j),

where θ*^j^*with elements $\theta_{0|1}^{j}$ and $\theta_{1|0}^{j}$ is the estimated parameter vector after receiving *j* inputs denoted by the vector **X***^j^*, the probability of the first stimulus is assumed to be $\frac{1}{2}$, and $n_{a|b}^{j}$ is the number of transitions from stimulus type *b* to stimulus type *a* in the *j* observations up to the present sample.

The parameter $n_{a|b}^{j}$ can be computed in different ways depending a forgetting model for the memory (Huettel et al., 2002; Kiebel et al., 2008; Harrison et al., 2011; Meyniel et al., 2016). In this paper, we have adopted a leaky integration method to account for earlier observations. In this method, the most recent stimulus is given a maximum weight and the weights of the preceding observations decrease exponentially with a parameter *w* (the integration coefficient) moving backward toward earlier observations (Meyniel et al., 2016).

Eq. 1 is the product of two Binomial distributions, each representing one of the two elements of the vectorθ. Using the Beta distribution notation to represent the prior probability of these elements as the conjugate prior of Binary distribution, the posterior distribution of θ*^j^* after *j* inputs will be the multiple of two new Beta distributions:


(2) $$p\,\left(\theta^{j}|{\text{X}}^{j}\right)=\text{Beta}\,\left(1+n_{0|1}^{j},1+n_{1|1}^{j}\right)\,\text{Beta}\,\left(1+n_{1|0}^{j},1+n_{0|0}^{j}\right),$$

To sum up, the posterior probability of the stimulus-generating Binomial distribution parameter is obtained using a two-dimensional descriptor parameter in Eq. 2. The next step is to use this equation to calculate the theoretical surprise inherent in the stimuli sequence.

### Surprise Calculation

In the previous section, we estimated the stimulus-generating distribution assuming transition probability matrix as sufficient statistics. When the brain encounters a stimulus that was not predicted using this estimated distribution, it may produce a “surprise” response reflecting the prediction error (Mars et al., 2008; Lieder et al., 2013; Meyniel et al., 2016; Rubin et al., 2016; Modirshanechi et al., 2019). There are three mathematical approaches in the literature to quantify this surprise. We elaborated the approaches and derived the formulas for calculating the three surprise measures completely in Supplementary Material. The labels of the decoder are these surprise values *Y*_*N×1*_ used to train the regressor.

### Temporal Analysis

Four methods for selecting the temporal components are employed as described below.

**FIGURE 2:**
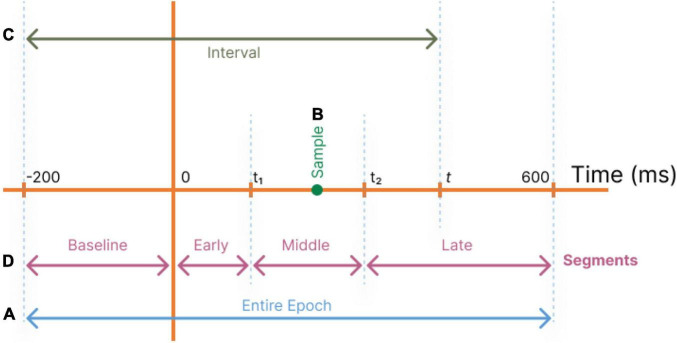
Different temporal component selection regimes are used to define feature vectors as inputs to the decoder. **(A)** All temporal components of a trial are used (Entire epoch). **(B)** Each single temporal point *t* is used (Samples). **(C)** Temporal components in the range of [−200,*t*] are used (Intervals). **(D)** Temporal segments are used with optimum *t*_*1*_ and*t*_2_ (Segments).

In our study, first we seek to identify the significant single time instances or time intervals, which can best regress the surprise value of the stimuli. Hence, we define four different regimes of selecting samples from the temporal data record (Figure 2):

1. **Entire epoch:** The total response time (-200 ms to 600 ms) is used for regression to identify all significant coefficients (Figure 2A).
2. **Samples:** A single sample at time *t*is employed as the decoder’s input (Figure 2B), and this operation is repeated for all values of *t* to determine their relative powers in estimating the stimuli surprise.
3. **Intervals:** To evaluate the significance of an interval of accumulated temporal samples from the beginning of the epoch to the current target time, the interval of −200 to time *t* is used as input to the decoder (Figure 2C). This operation is repeated for all values of *t*. This allows the decoder to utilize the dependency among the temporal samples in the recorded data.
4. **Segments (Baseline, Early, Middle, and Late):** To evaluate the regression power of the target time interval and to compare the segments of the MEG records, a range of temporal samples is used as the decoder’s input feature vector (Figure 2D).

Four disjoint time segments are identified for coarse-level segmentation of the response profile: From −200 ms pre-stimulus to time 0 (Baseline), from time 0 to *t*_*1*_ (Early components), from *t*_*1*_ to *t*_*2*_ (Middle components), and from *t*_*2*_ to 600 ms (Late components) (Figure 2D).

**FIGURE 3:**
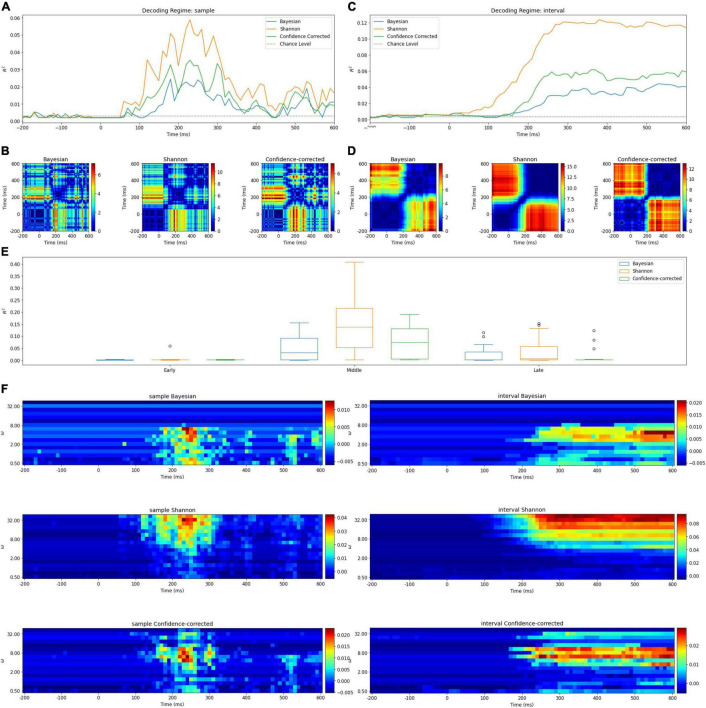
**(A)** Surprise decoding powers of the three surprise quantifications and chance level for the *Samples* regime. **(B)** Statistical analysis of the *Samples* regime. Each diagram illustrates an 80 × 80 matrix of *p*-values in logarithmic scale obtained by *t*-test. The red areas indicate the presence of significant differences between the two samples representing the horizontal and vertical axes values. **(C)** Surprise decoding powers of the three surprise quantifications and chance level for the temporal regime of *Intervals* [-200 ms, *t*]. **(D)** Statistical analysis of the *Intervals* regime. Each diagram illustrates an 80 × 80 matrix of *p*-values in logarithmic scale obtained by *t*-test to evaluate the significance of difference between decoding powers of each pair of features in the *Intervals* regime. **(E)** Box plots of decoding powers for different *Segments* (early, middle, and late). In these boxplots, horizontal lines indicate the median of the data and the boxes extend from the lower to upper quartile values. The whiskers extending from the boxes indicate the range of the data. Flier points are those past the ends of the whiskers. **(F)** The decoding power of different values of *w* (vertical axis) over time (horizontal axis) for different definitions of surprise in the *Intervals* and *Samples* regimes. Colors denote the decoding power. Note that the *p*-values are shown in a logarithmic scale for better visualization.

In our work, the values of *t*_*1*_ and *t*_*2*_ are determined to provide decoding behavior-based definitions for the Early, Middle, and Late segments using the decoding powers obtained in the *Samples* regime. After analyzing the *Samples* regime, we define *t*_*1*_ as the first point that reaches the 10 percent of the globally maximum decoding power. Furthermore, observing that two local maxima exist in the middle and late responses, we define *t*_*2*_ as the point with minimum decoding power in the interval [250 ms, 400 ms] in order to separate the Middle and Late segments (see Figure 3A in Section “Temporal Analysis”). When we capitalize the name of these segments, we mean the segments with boundaries defined based on this approach.

### Spatial Analysis

**FIGURE 4:**
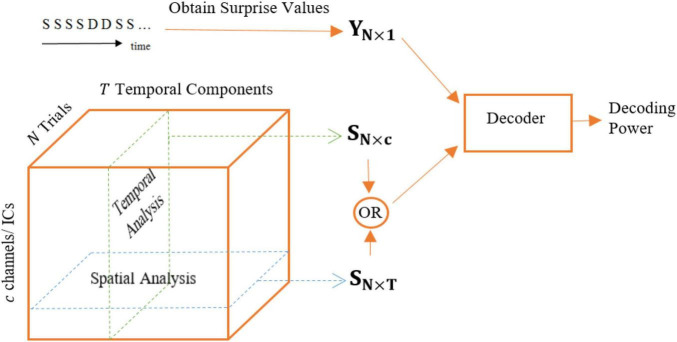
Different feature matrices used for temporal (*Samples* regime) and spatial analysis: *S*_*N×c*_ and *S*_*N×T*_, respectively.

Spatial analysis is performed in an essentially similar fashion to the temporal analysis but the feature matrix is defined in such a way to allow for comparing the different magnetometers in collecting the most surprise-correlated brain activity (see Figure 4). Similarly, the decoding model is essentially a Lasso linear regression module and the labels for this regressor are the calculated theoretical surprise values. We perform two methods of analysis for the data of each channel (magnetometer):

1. The feature matrix fed to the regression module is an *N*×*T* matrix, i.e., all temporal samples are used to decode the level of surprise for each magnetometer.
2. The feature matrix fed to the regression module is an *N*×*T*′ matrix, where *T*′ < *T*, meaning that a portion of the temporal samples is used to regress the level of surprise. The goal is adding a temporal view to the spatial analysis in order to compare the surprise-decoding regions on the scalp using different temporal segments: Early, Middle, and Late segments.

### The Decoder Design

The decoder we use for this analysis was introduced by Modirshanechi et al. (2019), and we modified its input features as well as the surprise labels to fit our analysis as described above. More details about the decoder can be found in Modirshanechi et al. (2019).

Briefly, the decoder mainly consists of one module of linear regression. A Lasso linear regression model takes as its input the feature matrix*S*_*N*×*p*′_ extracted from the data according to one of the 4 described temporal feature selection regimes (*N* is the number of features and *p*′≤*p* is the dimension of each feature which depends on the temporal feature selection regime), as well as the label vector *Y*_*N×1*_ calculated from the input stimuli sequence according to one of the three mentioned definitions of surprise as its labels (see Figure 1). The Lasso regressor aims to minimize the reconstruction error while observing an added sparsity term, eliminating the input features that might be irrelevant to the reconstruction of surprise, and helps avoid overfitting to the training data.

To evaluate the trained model on the test data using a fivefold cross-validation, we used the R-squared measure as decoding power. These values were compared to chance levels to test (and reject) the hypothesis that the input features are independent from surprise labels. Noticing that the decoding power is a function of the integration coefficient *w* (the parameter defining the coefficients of the window of integration), we reported the maximum decoding power across all the *w* values for each regression by employing the best integration coefficients. Also, in the end, we reported and analyzed the best values for the integration coefficients averaged over subjects. After the removal of features with zero coefficients by the Lasso regressor, the remaining features were presumed effective and employed in describing the surprise.

At the end of each decoding analysis task, to judge the resulting *R*-squared values, we tested the hypothesis that *S*_*N×p*_ and *Y*_*N×1*_are independent of each other (Rouder et al., 2009; Modirshanechi et al., 2019). This was done by making random permutations in the vector *Y*_*N×1*_ and acquiring the *R*-squared value of the resulting regression each time as chance level (Pereira et al., 2009).

The entire analysis was performed separately for each subject and for each type of surprise. We used Matlab to design and simulate the decoder.

## Results

**FIGURE 5:**
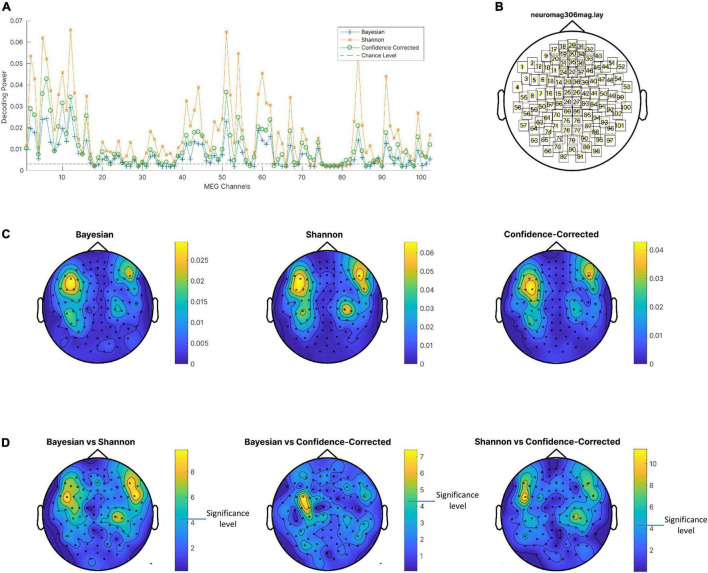
**(A)** Decoding power of magnetometers averaged on subjects for the three surprise values and chance level. **(B)** Channel locations. **(C)** Decoding powers of three surprise values in different channels using the entire response epoch. **(D)** *P-value* of *t*-test of difference between decoding powers of pairs of surprise values. Channels with lower *p*-values are shown in yellow. Note that a different color scale is used in each plot for better visibility of areas with high decoding powers. Also, *p*-values are logarithmically scaled for better visualization.

**FIGURE 6:**
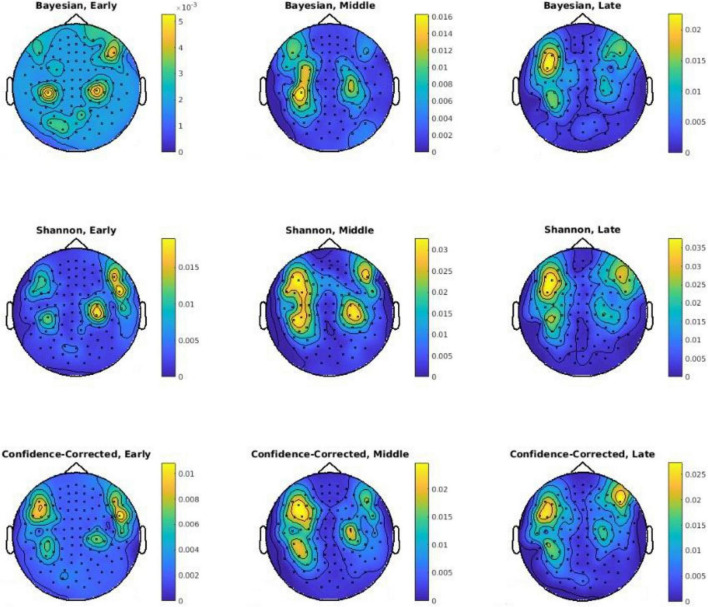
Sensor-level topographies of decoding powers of three surprise values in different channels using the Early, Middle, and Late segments. Note that a different color scales is used in each plot for better visibility of areas with high decoding powers. Also, *p*-values are logarithmically scaled for better visualization.

Tables 1–4 and Figures 3, 5, 6 summarize our results, which are described in detail next.

**TABLE 1:** Decoding power (*R*^2^ values), chance level, and *p*-values of *t*-tests comparing chance levels and decoding powers for the three definitions of surprise for the temporal regime of *Entire epoch*.

Decoding power	Shannon	Confidence-corrected	Bayesian
Entire epoch	0.134	0.070	0.033
**Chance level**			
Entire epoch	−0.0031 ± 0.0050	−0.0031 ± 0.0051	−0.0031 ± 0.0050
***p*-values**			
Entire epoch	**0.000100**	**0.000112**	**0.000747**

*Highlighted p-values are the ones lower than significance level using Bonferroni correction (equal to 0.0042).*

**TABLE 2:** The temporal borders separating the Early, Middle, and Late segments obtained from partitioning the decoding power curves in Figure 3A.

	Shannon	Bayesian	Confidence-corrected
(*t*_*1*_, *t*_*2*_) (see Figure 2D)	(60, 350)	(50, 360)	(60, 380)

**TABLE 3:** Decoding power, chance level, and *p*-values of *t*-tests comparing chance levels and decoding powers for the three definitions of surprise for the temporal regime of *Segments*.

Decoding power	Shannon	Bayesian	Confidence-corrected
Early	0.004 ± 0.007	0.002 ± 0.001	0.002 ± 0.001
Middle	0.147 ± 0.116	0.056 ± 0.066	0.087 ± 0.088
Late	0.036 ± 0.046	0.025 ± 0.035	0.021 ± 0.038
**Chance level**			
Early	−0.0031 ± 0.0050	−0.0031 ± 0.0051	−0.0031 ± 0.0051
Middle	−0.0031 ± 0.0050	−0.0031 ± 0.0051	−0.0031 ± 0.0051
Late	−0.0031 ± 0.0050	−0.0004 ± 0.0069	−0.0031 ± 0.0051
***p*−values**			
Early	0.015457	**0.000160**	**0.000112**
Middle	**0.000001**	**0.000228**	**0.000035**
Late	**0.002373**	0.005639	0.024679

*Highlighted p-values are the ones lower than significance level using Bonferroni correction (equal to 0.0042).*

**TABLE 4:** Results of the ANOVA test for comparing three temporal segments of Early, Middle, and Late of the *Segments* regime in decoding each surprise.

Surprise model	ANOVA *f*-value	*p*-value (Middle vs. Late)	*p*-value (Early vs. Middle)	*p*-value (Early vs. Late)
Shannon	18.909	**0.0007**	**1.31e-05**	**0.0079**
Bayesian	7.575	0.0919	**0.0017**	**0.0106**
Confidence-corrected	11.937	**0.0077**	**0.0003**	0.0501

*Highlighted p-values are the ones lower than significance level using Bonferroni correction (equal to 0.0167).*

### Temporal Analysis

Here we describe the decoding powers of the three quantifications of surprise when each is employed as label for training surprise decoders.

#### Entire Epoch

The *R*^2^ values and chance levels when using the entire epoch are presented in Table 1. The mean of *R*^2^ values are at least ten times bigger than the mean of chance levels. We conducted *t*-tests to examine the presence of significant differences between chance levels and decoding powers. We corrected the significance level using Bonferroni correction (Bonferroni, 1936) considering 12 tests to 0.0042 (we conducted these tests with the tests of Table 3 that compares decoding powers with the chance levels for the Segments simultaneously. Three surprise models and four segments lead to 12 tests). We observed that the decoding powers are significantly higher than chance level in the *Entire epoch* regime. The values of chance level and decoding power in this table can be considered as upper bounds for other goodness of fit measurements in the other three temporal regimes. We selected the maximum value among the three chance levels to plot for comparison in Figures 3A,C,5A.

#### Samples Regime

The decoding power of this regression model is illustrated in Figure 3A for different values of *t* ∈ [−200ms,  600ms]. Due to the employment of only one time sample in each epoch for describing the trial’s surprise; it is understandable to have relatively low *R*^2^ levels. In the curves of Figure 3A, the middle and late components appear to describe surprise better than the early components. In addition, one noticeable peak is observed in the middle segment. The fact that the *Samples* regime is able to identify time points in the middle segment of the MEG response with the highest surprise-decoding powers (for any of the three definitions of surprise) is a remarkable observation in our study.

#### Intervals Regime

Figure 3C illustrates the decoding powers of decoders trained using an interval of temporal samples in the range of [-200 ms, *t*] for different values of *t*. This regime is expected to reveal at which time instance enough evidence has been accumulated from the response for achieving a confident decoding performance. In each curve, the *R*^2^ value stays close to zero until around 100 ms, when there is a considerable rise in the decoding power. This increase occurs in the temporal range which we called the middle segment in our *Segments* regime. The decoding power increases only little after around 250 ms. We can deduct that the response components do not add much information about surprise after around 250 ms.

#### Segments Regime

First, the time points that best partition the entire after-onset epoch to three parts are obtained based on the method described in Section “Preprocessing of Data” and reported in Table 2.

The *R*^2^ values and chance levels for using data points in each of the segments named Early, Middle, and Late for decoding surprise are presented in Table 3. We observe that for the Early segment, the *R*^2^ values and the chance levels are close to each other. We conducted *t*-tests to examine the presence of significant differences between chance levels and decoding powers. We corrected the significance level using Bonferroni correction to 0.0042. We observed that decoding powers are significantly higher than chance level in the Middle segment for all three surprise models. However, we did not observe this significance for the Early and Late segments in any of the surprise models. This result is expected since the early segment of the response epoch is known to have little or no information about surprise and has been reported to mainly reflect the physical aspects of the stimuli (Sur and Sinha, 2009). To explain this result, we note that even though the characteristics of the two types of stimuli (standards and deviants) are different from each other, the components recorded during the early processing of the stimuli do not appear to account for the stimuli’s surprise. In other words, these processes also seem to create signatures in the recorded response that are not differentiable from each other in a significant way as far as the issue of their confound with the brain’s surprise is considered. The latter point is a remarkable observation which our statistical analysis also reveals and as such, provides further evidence that early sensory processes in the brain employ generic sets of operations on all stimuli as the surprise aspects of the input are still not known to the brain.

We can further add that even though the differences between the characteristics of the two types of stimuli may affect the early part of the recorded brain response (which might be observed as differences between the two responses when the usual trial averaging techniques are used and decoding the surprise of each trial is not an objective), such differences in the recorded response cannot be used to decode the surprise that is embedded in the input sequence. In other words, this lack of differentiability in terms of surprise decoding between the early parts of the response to the stimuli can itself serve as an indication that the input characteristics do not interject any confound into the decoding process employed in our model.

We observe in Table 3 that the Middle segment demonstrates significant values of decoding power. Figure 3E shows the variation of the decoding powers of different segments across the subjects using three boxplots for the three surprise values.

#### Significance of Temporal Features

Table 4 shows the results of repeated measures of ANOVA (Analysis of Variance) (Gueorguieva and Krystal, 2004) for comparing the decoding powers of the three segments of Early, Middle, and Late, employing data from the different subjects as the statistical samples. The *f*-value of the ANOVA analysis and the *p*-values of the *post hoc* analysis are reported in the table. We conducted three one-way ANOVA tests each corresponding to a surprise model. The significance level is corrected to 0.0167 using Bonferroni correction.

The table indicates that not only the Early segment is significantly less powerful than the Middle segment, but also significant difference is observed between the *R*^2^ of the Shannon and Confidence-corrected surprise values for the Middle and Late segments. This is because the Late components, as it was also observed in the results of the *Samples* regime, are significantly less powerful than the Middle components. However, this is not the case for the Bayesian surprise, which offers relatively similar decoding powers for the Middle and Late segments.

Similarly, in Figure 3B, the relative importance of temporal components for decoding surprise is assessed for decoders based on the *Samples* regime. In these figures, each picture illustrates an 80×80matrix of −*log*_2_(*p*−*values*) coded to colors, representing *1600* tests performed to evaluate the significance of the difference between the decoding powers of each pair of features in the *Samples* regime. We used the logarithmic *p*-value scale to afford a wider range for better visualization. Note that these are uncorrected *p*-values as we only want to compare the relative levels of *p*-values here and scaling all of them (using Bonferroni correction) has no impact. A similar plot is shown in Figure 3D for the *Intervals* regime.

In the *Samples* regime, there is no single time instance with significantly better decoding power (for decoding any of the three surprise quantifications) than all the other temporal points (Figure 3B).

In the *Intervals regime*, the relatively narrow diagonal blue line around the point (200 ms, 200 ms) shows the rapid rising behavior of the decoding power when the points of the middle segment are included (Figure 3D). Furthermore, after around 250 ms (in Figure 3D), adding new temporal components as features for decoding surprise (for any of the three definitions) does not lead to a significantly higher decoding power.

#### The Effect of Integration Coefficient

In Figure 3F, the decoding powers of the designed decoders are plotted for different integration coefficients in the range of [1,100]. Two different behaviors can be observed for the three surprise quantifications. For the Shannon and confidence-corrected surprise values, when *w* is not small, a relatively high decoding power is observed. However, for the Bayesian surprise *w* needs to be relatively small in order to obtain high decoding powers.

In addition, in the *Samples* regime of this analysis, the best integration coefficient is not much dependent on time. In other words, the best *w* is not much different for the middle and late components (Figure 3F).

### Spatial Analysis

In this part, first the decoding power for each of the 102 magnetometers is obtained for the three surprise quantifications using the entire temporal epoch as the input feature for regressors. In Figure 5A the decoding power is averaged over subjects and plotted for all channels. The value of the decoding power is clearly greater in comparison to the chance level listed in Table 1, so the assumption of independence between surprise values and the entire epoch of the MEG data can be rejected. Interestingly, for almost all magnetometers, the MEG data decodes Shannon surprise best and Bayesian surprise worst. However, these comparisons are also statistically assessed using paired *t*-test to see whether the difference of decoding powers between pairs of surprise values is significant for each channel considering the subjects as samples. The resulting *p*-values are plotted as topographic maps in Figure 5D with lower *p*-value shown in yellow. The *p*-values are uncorrected and shown in logarithmic scale for better visualization. These plots are produced using the FieldTrip toolbox (Oostenveld et al., 2011) on the “neuromag306mag” layout, which is shown in Figure 5B. Then, the average values of the decoding power over the subjects are plotted as topographic maps in Figure 5C in which the brighter channels are the best magnetometers that can be selected for decoding Shannon (middle), Bayesian (left), and confidence-corrected (right) surprise values.

In the second part of the analysis, the decoding power of each channel is assessed temporally for the three defined segments of Early, Middle, and Late (see Figure 2). The goal is to gain an insight into the spatiotemporal value of the data in terms of describing surprise. Figure 6 depicts topographic plots of decoding powers for the three surprise quantifications for each of the mentioned temporal segments. The Middle segment possesses the highest level of decoding power, and the Late segment offers a lower decoding power compared to the Middle segment. These topographies in the Early and Late segments include the areas reported by Wacongne et al. (2011) for the effect of local mismatch at 120 ms and the effect of global deviance at 350 ms after the onset. Local mismatch and global deviance can lead to high theoretical surprise and relate the temporal samples reported by Wacongne et al. (2011) to our results. In addition, Strauss et al. (2015) reported the effect of local mismatch at 150 ms and the global variance at 350 ms for MEG data, which are correlated with the temporal segments used to decode surprise in the Middle and Late segments in our analysis.

## Discussion

### Evidence for Bayesian Brain and Ideal Observer

The assumptions of the Bayesian brain and the ideal observer (Knill and Pouget, 2004; Behrens et al., 2007; Mathys et al., 2011; Nassar et al., 2012, 2010) are embedded in the way we have calculated the theoretical surprise of each stimulus. Although there are three different approaches for defining this surprise, all are based on the parameters learned following the Bayesian brain and ideal observer assumptions. Our results demonstrate the feasibility of decoding these three quantifications of the theoretical surprise on a trial-by-trial basis with significant decoding power, and hence provide new evidence for supporting the Bayesian brain and ideal observer assumptions.

### Optimal Use of Temporal Components in Measuring Surprise

A remarkable distinction of our work is that we have not considered any single predefined temporal sample as a representative for the surprise of the brain. Extracting a reliable single temporal value from each epoch (even after epoch averaging, which is a common practice in ERP analysis) is a complex and rather *ad hoc* procedure (Debener et al., 2005; Cecotti and Graser, 2010; Turnip et al., 2011; Amini et al., 2013; Kolossa et al., 2013). In our approach, we use data from the entire response on a trial-by-trial basis to derive the surprise of the brain as a linear combination of the samples of the response with optimally determined weights.

### Optimal Use of the Spatially Distributed Effects of Surprise

Earlier studies based on fMRI data analysis have reported that the Shannon and Bayesian surprise values are modulated in different brain regions (Ostwald et al., 2012; O’Reilly et al., 2013; Schwartenbeck et al., 2016; Visalli et al., 2019). In addition, the well-known surprise-related components of the ERP signal such as MMN and P300 have been shown to emanate from the fronto-parietal and the fronto-central regions of the brain, respectively (Giard et al., 1990). In the current study, we have not imposed any spatial preferences between the magnetometers or among the ICs with spatial distributions close to the known sources of surprise in the brain. This choice offers generality to our analysis through employing all available data and letting the decoders capture all the relevant information during the training procedure. In fact, we employed a sparse regression model, which forces the coefficients of the surprise-irrelevant temporal/spatial components to be zero.

### Optimizing the Timescale of Integration

The best description for the Bayesian surprise derived from the brain’s response occurs when a rather short window of integration is used. This behavior stems from the very definition of the Bayesian surprise. The value of the KL divergence constantly decreases as we increase the timescale of integration since the two distributions involved become closer to each other. Given the rather short window of integration involved in keeping track of the Bayesian surprise, this quantification of surprise tends to be more sensitive to fluctuations in the recorded data compared to the Shannon and confidence-corrected surprises. The latter two use longer windows of integration, and are hence more robust to such fluctuations and can provide more accurate estimates of the underlying statistics of the input sequence generation process. This superiority is reflected in the higher decoding performance for these two concepts of surprise over the Bayesian surprise as illustrated across all of our results.

### Magnetoencephalography and Electroencephalography Comparison

Malmivuo (2012) suggested that MEG and EEG recordings are only partially independent. While EEG-based studies have provided an understanding of the temporal and spatial signatures of surprise, the better signal-to-noise ratio and readability of the MEG recordings compared to EEG (Hämäläinen et al., 1993; Strauss et al., 2015) offer opportunities based on MEG data for further examination of the mechanisms that generate surprise in the brain. A larger number of recording sensors distributed more densely across the head, as is often the case for MEG recordings, provides better coverage of local activity beneath the scalp.

A likely explanation for the lower performance of the late components in the MEG analysis in our decoding model, which do not reflect the powerful P300 response in EEG data, can be that while each EEG sensor collects and integrates data from a rather distributed and deep set of sources in the brain (Malmivuo and Plonsey, 1995), each MEG sensor can only capture the activities of sources in its close proximity beneath the scalp (Schomer and Da Silva, 2012). The surprise generation mechanisms of the brain transmit signals to a number of different regions of the brain, which in turn produce the late components of surprise which are distributed and diffused. The relatively lower decoding power of the late components in MEG records can be explained by noting that since these late components are generated by distributed sources, MEG sensors may not be able to adequately capture them (Wacongne et al., 2011; Ilmoniemi and Sarvas, 2019).

### Spatial Signatures of Surprise

Frontal regions of the cortex (including the dorsal cingulate cortex) were reported in fMRI studies (e.g., by Schwartenbeck et al., 2016) to modulate activities related to information-theoretic (Shannon) surprise. The posterior parietal cortex (O’Reilly et al., 2013) and the inferior frontal gyrus are proposed as two regions that correlate with both the Shannon and Bayesian surprises (Visalli et al., 2019). Our observations on data collected from the scalp by MEG sensors are in agreement with these fMRI-based studies. Magnetometers placed on the two sides of the frontal midline may detect the surprise-related activity of the dorsal cingulate cortex, which is located closer to the scalp, while magnetometers placed on the two sides of the parietal midline may detect the activities of the posterior parietal cortex (see Figures 5, 6). However, making interpretations about the sources evoked by auditory stimulation which result in such topographic maps is subject to ambiguity as discussed in the literature for some time (Hämäläinen et al., 1995). On the one hand, interpretations such as above may be challenged in light of the implied orientation of the underlying sources, i.e., to have both the cingulate and posterior parietal source dipoles be oriented along the anterior-posterior axis, which is not expected anatomically. Accordingly, an alternative interpretation of the topographic maps in Figures 5, 6 could be that they might reveal activities corresponding to bilateral superior temporal lobe sources as maps similar to those are typically evoked by auditory stimuli and are reported to indicate bilateral auditory cortex sources (Zevin, 2009). On the other hand, some studies argue for not attributing MEG sources to deep regions of the brain (like temporal lobe) by pointing out that the MEG data acquisition is most sensitive to superficial sources, and that its sensitivity is much reduced for deep sources (Cohen and Cuffin, 1983; de Jongh et al., 2005; Ahlfors et al., 2010). According to such observations, attributing the four maxima in the maps of Figures 5, 6 to frontal and parietal source pairs may be a possibility. However, and adding to the complexity of making interpretations on the MEG topographical maps, one could also mention the possibility that bilateral sources in the auditory cortices may also produce an extra deflection in these maps close to the posterior midline due to the proximity of fields from the two sources which have opposite directions (Hämäläinen et al., 1995).

## Conclusion

Surprise and its impact have been well characterized in many studies on social interactions as well as in computational frameworks using recorded brain signals. However, an information-theoretical model to describe and predict the surprise level of an external stimulus in recorded MEG data has not been reported to date. The current study proposed a regression model for decoding the level of the brain’s surprise in response to sensory sequences using optimally selected temporal components of recorded MEG data. Three surprise quantification definitions, Shannon, Bayesian, and confidence-corrected, were assessed in offering decoding power in modeling the recorded data. Four different regimes for selecting temporal samples were used to evaluate which parts of the recorded data contain signatures that best represent the brain’s surprise. We found that the middle temporal components of the MEG response offer the strongest power for decoding surprise. The best magnetometers for collecting the activities related to all three concepts of surprise were found to be in the right and left fronto-central regions. Measuring surprise of the brain by decoding techniques such as the method proposed in the current study can complement data obtained *via* behavioral observations in order to devise computational models for evaluating the effect of surprise in social interactions.

## Data Availability Statement

Publicly available datasets were analyzed in this study. This data can be found here: https://osf.io/wtnke/.

## Author Contributions

MK and ZM contributed to material preparation and data analysis. ZM wrote the first draft of the manuscript. HA supervised the work and edited the manuscript. All authors contributed to the study conception and design, read, and approved the final manuscript.

## Conflict of Interest

The authors declare that the research was conducted in the absence of any commercial or financial relationships that could be construed as a potential conflict of interest.

## Publisher’s Note

All claims expressed in this article are solely those of the authors and do not necessarily represent those of their affiliated organizations, or those of the publisher, the editors and the reviewers. Any product that may be evaluated in this article, or claim that may be made by its manufacturer, is not guaranteed or endorsed by the publisher.
