# Supplementary material for: Spatiotemporal Signatures of Surprise Captured by Magnetoencephalography
Source: Front Syst Neurosci. 2022 Jun 13;16:865453. doi: 10.3389/fnsys.2022.865453 (PMC9235820; doi:10.3389/fnsys.2022.865453)
Supplement: Supplementary file 1 [file Data_Sheet_1.docx]

Supplementary Material

## 1. Surprise Definition and Calculation

In our decoding model, surprise values $Y_{N\times1}$ are employed as labels in the process of training the decoder (Fig. S1).


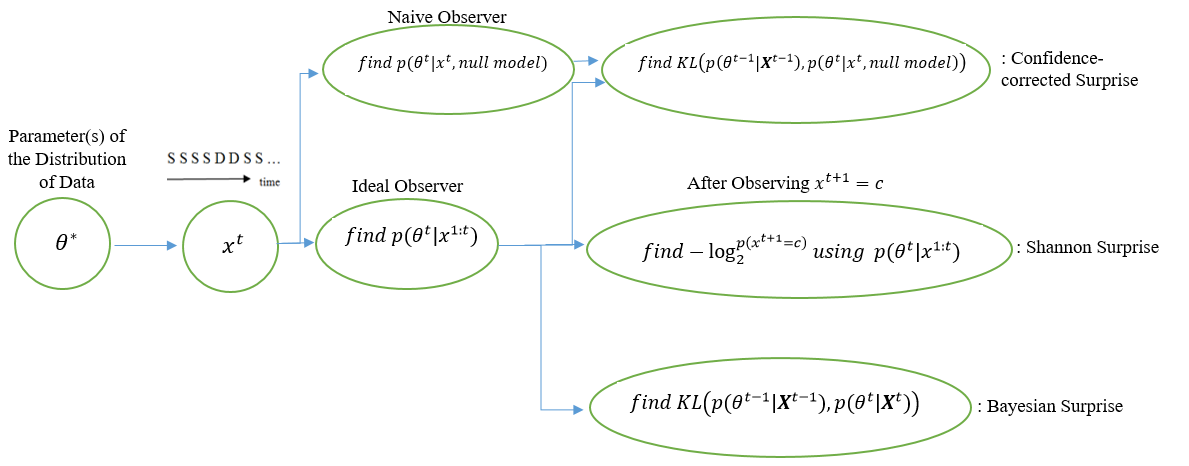


**Figure S1.** Calculating the three different quantifications of surprise.

Estimation of the stimulus-generating distribution leads to a prediction about the next stimulus, which if violated may produce a “surprise” response by the brain, reflecting the prediction error (Lieder et al., 2013; Mars et al., 2008; Meyniel et al., 2016; Modirshanechi et al., 2019; Rubin et al., 2016).

## 1.1. Shannon Surprise

Shannon surprise provides a measure of surprise based on the predicted probability for an observed event (Meyniel et al., 2016; Modirshanechi et al., 2019; Shannon, 1948; Strange et al., 2005). The more unlikely an observation is, the more surprising it will be. Shannon surprise can be computed for the case of the $j^{th}$stimulus having a value$c$:

$$Shannon surprise\left( x^{j}=c \right)= -{\log_{2}\hat{p}}_{c}, (3)$$

where c is 0 or 1 in an oddball test and $\hat{p}_{c}$ is the predicted probability of stimulus $j$ to be $c$ and is obtained using this formula:

$$\hat{p}_{c}=\int p(x^{j}=c\left| \boldsymbol{\theta}^{j-1} \right)p\left( {\boldsymbol{\theta}^{j-1}\boldsymbol{|X}}^{j-1} \right)d\boldsymbol{\theta}^{j-1}$$

It can be shown that the probability of the $j^{th}$ item having a value of 0 or 1 can be estimated using the estimated value of $\boldsymbol{\theta}$ at the preceding time (i.e.$\boldsymbol{\theta}^{j-1}= {[\theta_{0|1}^{j-1}\theta_{1|0}^{j-1}]}^{T}$) via:

$${p(x}^{j}=0\left| \boldsymbol{\theta}^{j-1} \right)=\frac{{{\theta_{0|1}^{j-1}}}}{{(\theta}_{0|1}^{j-1}+\theta_{1|0}^{j-1})}$$

$${p(x}^{j}=1\left| \boldsymbol{\theta}^{j-1} \right)=\frac{\theta_{1|0}^{j-1}}{{(\theta}_{0|1}^{j-1}+\theta_{1|0}^{j-1})}$$

## 1.2. Bayesian Surprise

The Bayesian surprise measures how much difference is induced in the estimated generative distribution after observing each stimulus. In fact, this surprise can be calculated using the Kullback–Leibler (KL) divergence of two distributions assessed before and after observing each observation (Cover, 1999; Kullback, 1997). For the two-dimensional $\boldsymbol{\theta}$ parameter, the surprise after observing $j^{th}$ stimulus is:

$$Bayesian surprise\triangleq\mathrm{KL}\left( p\left( {\boldsymbol{\theta}^{j-1}|\boldsymbol{X}}^{j-1} \right),p\left( {\boldsymbol{\theta}^{j}|\boldsymbol{X}}^{j} \right) \right), \left( 4 \right)$$

which is the distance between the distribution estimated right before the arrival of stimulus *j* based on the previous $j-1$ observations, and the distribution estimated after the arrival of stimulus *j*.

## 1.3. Confidence-corrected Surprise

Assume a choice reaction time task with four types of stimuli [$A,B, C, D]$ creates two models of the world for two observers. The first observer believes that all stimuli types are equally likely (i.e. the probabilities of the four types are [0.25, 0.25, 0.25, 0.25]), while the second observer estimates the distribution as [0.75, 0.25, 0, 0]. After receiving an input *B*, both observers will have the same puzzlement surprise equal to $-\log_{2} 0.25$= 2 in the Shannon’s model of surprise. But it is obvious that the first person is surprised less than the second one due to his indifference to the predicted probability of occurrence between all types.

This most recent definition for surprise, being calculated in the puzzlement sense, considers how observers commit to their belief about the world, and attempts to address the issue reflected in the above example by considering not only the estimated probability of the current value of the input, but also the estimated probability of other possible values of the input (Faraji et al., 2018). It measures the distance between the distribution estimated for model parameters after observing all the $j-1$ samples, and the posterior belief of a naїve observer for parameters after observing the$j^{th} sample,x^{j}$. The latter is indeed the posterior probability of parameters considering a uniform prior, after receiving $x^{j}$as the first observation. Therefore,

$$confidence-corrected surprise= \mathrm{KL}\left( p\left( {\boldsymbol{\theta}^{j-1}|\boldsymbol{X}}^{j-1} \right),p\left( {\boldsymbol{\theta}|\boldsymbol{x}}^{j}, uniform prior belief \right) \right), (5)$$

where $\boldsymbol{\theta}$ can be a number or a vector. For the binary oddball paradigm case, this can be modeled as:

$p\left( {\boldsymbol{\theta}|\boldsymbol{x}}^{j}, uniform prior belief \right)= \mathrm{Beta}(1+I_{0}^{j},1+I_{1}^{j})$*,*

where $I_{k}^{j}$ is the identity function ($I_{k}^{j}$ is one if $x^{j}=k$ , and zero otherwise) in the single-parameter model. In the two-parameter transition matrix model:

$$p\left( {\boldsymbol{\theta}|\boldsymbol{x}}^{j}, uniform prior belief \right)=$$

$\mathrm{Beta}\left( 1+{I^{j}}_{0|1},1+{I^{j}}_{1|1} \right)\mathrm{Beta}\left( 1+{I^{j}}_{1|0},1+{I^{j}}_{0|0} \right)$*,*

with $I_{k|l}^{j}$defined as one if $x^{j}=k$and $x^{j-1}=l$, and zero otherwise.

Each of these three definitions of surprise yields a vector $Y_{N\times1}$, with elements to be used as surprise labels for each of the $N$ trials in training a surprise decoder. The analysis is performed for a binary oddball task in the form of decoding surprise responses, and comparisons between the predictive powers of different temporal components/segments are made based on the two-parameter model described earlier.

**References**

Cover, T. M. (1999). Elements of information theory. John Wiley & Sons.

Faraji, M., Preuschoff, K., & Gerstner, W. (2018). Balancing new against old information: the role of puzzlement surprise in learning. Neural computation, 30(1), 34-83.

Kullback, S. (1997). Information theory and statistics: Courier Corporation.

Lieder, F., Daunizeau, J., Garrido, M. I., Friston, K. J., & Stephan, K. E. (2013). Modelling trial-by-trial changes in the mismatch negativity. PLoS Comput Biol, 9(2), e1002911.

Mars, R. B., Debener, S., Gladwin, T. E., Harrison, L. M., Haggard, P., Rothwell, J. C., & Bestmann, S. (2008). Trial-by-trial fluctuations in the event-related electroencephalogram reflect dynamic changes in the degree of surprise. Journal of Neuroscience, 28(47), 12539-12545.

Meyniel, F., Maheu, M., & Dehaene, S. (2016). Human inferences about sequences: A minimal transition probability model. PLoS computational biology, 12(12), e1005260.

Modirshanechi, A., Kiani, M. M., & Aghajan, H. (2019). Trial-by-trial surprise-decoding model for visual and auditory binary oddball tasks. NeuroImage, 196, 302-317.

Rubin, J., Ulanovsky, N., Nelken, I., & Tishby, N. (2016). The representation of prediction error in auditory cortex. PLoS computational biology, 12(8), e1005058.

Shannon, C. E. (1948). A mathematical theory of communication. The Bell system technical journal, 27(3), 379-423.

Strange, B. A., Duggins, A., Penny, W., Dolan, R. J., & Friston, K. J. (2005). Information theory, novelty and hippocampal responses: unpredicted or unpredictable? Neural Networks, 18(3), 225-230.
